# Thermal Conductivity and Sintering Mechanism of Aluminum/Diamond Composites Prepared by DC-Assisted Fast Hot-Pressing Sintering

**DOI:** 10.3390/ma17091992

**Published:** 2024-04-25

**Authors:** Jianping Jia, Xiaoxuan Hei, Xiao Yang, Wei Zhao, Yuqi Wang, Qing Zhuo, Yuanyuan Li, Hangyu Dong, Futian Liu, Yingru Li, Xiaoshan Yan

**Affiliations:** 1 Faculty of Science, Yibin University, Yibin 644007, China; 2 School of Materials Science and Engineering, University of Jinan, Jinan 250024, China; heixx1125@163.com (X.H.); mse_liuft@ujn.edu.cn (F.L.); 3 College of Intelligent Systems Science and Engineering, Hubei Minzu University, Enshi 445000, China; yancy2023@outlook.com (X.Y.); 202230510@hbmzu.edu.cn (W.Z.); 202230511@hbmzu.edu.cn (Y.W.); 202330293@hbmzu.edu.cn (Q.Z.); 2023026@hbmzu.edu.cn (Y.L.); 2022011@hbmzu.edu.cn (H.D.); 2020030@hbmzu.edu.cn (Y.L.); 4 Key Laboratory of Green Manufacturing of Super-Light Elastomer Materials of State Ethnic Affairs Commission, Hubei Minzu University, Enshi 445000, China; 5 State Key Laboratory of NBC Protection for Civilian, Research Institution of Chemical Defense, Beijing 100191, China

**Keywords:** Al/diamond composites, thermal conductivity, sintering mechanism, DC-assisted fast hot-pressing sintering, packaging material

## Abstract

A novel DC-assisted fast hot-pressing (FHP) powder sintering technique was utilized to prepare Al/Diamond composites. Three series of orthogonal experiments were designed and conducted to explore the effects of sintering temperature, sintering pressure, and holding time on the thermal conductivity (TC) and sintering mechanism of an Al-50Diamond composite. Improper sintering temperatures dramatically degraded the TC, as relatively low temperatures (≤520 °C) led to the retention of a large number of pores, while higher temperatures (≥600 °C) caused unavoidable debonding cracks. Excessive pressure (≥100 MPa) induced lattice distortion and the accumulation of dislocations, whereas a prolonged holding time (≥20 min) would most likely cause the Al phase to aggregate into clusters due to surface tension. The optimal process parameters for the preparation of Al-50diamond composites by the FHP method were 560 °C-80 MPa-10 min, corresponding to a density and TC of 3.09 g cm^−3^ and 527.8 W m^−1^ K^−1^, respectively. Structural defects such as pores, dislocations, debonding cracks, and agglomerations within the composite strongly enhance the interfacial thermal resistance (ITR), thereby deteriorating TC performance. Considering the ITR of the binary solid-phase composite, the Hasselman–Johnson model can more accurately predict the TC of Al-50diamond composites for FHP technology under an optimal process with a 3.4% error rate (509.6 W m^−1^ K^−1^ to 527.8 W m^−1^ K^−1^). The theoretical thermal conductivity of the binary composites estimated by data modeling (Hasselman–Johnson Model, etc.) matches well with the actual thermal conductivity of the sintered samples using the FHP method.

## 1. Introduction

With the advancement of semiconductor and microelectronics industries, high-performance electronic components with increasing power densities continue to emerge, which pose new challenges to the heat dissipation performance of thermal management materials [1,2,3,4]. The thermal dissipation performance of microelectronic devices is primarily limited by the packaging material. An ideal electronic packaging material should have a relatively high thermal conductivity (>200 W m^−1^ K^−1^) in order to maximize heat dissipation to keep the microelectronic devices at a relatively low temperature [5]. With the increase in heat generated by advanced microelectronic devices, conventional packaging materials, including Al, Cu, Cu-W, C-Mo, Kovar alloys, etc., gradually become unable to meet the requirements of increasingly advanced electronic applications [4,6,7]. Therefore, researchers have devoted themselves to preparing metal matrix composites (MMCs) by adding thermally conductive reinforcements to the original high thermal conductivity matrix to further enhance the thermal conductivity (TC).

Aluminum has been widely applied as a matrix for composite materials due to its large reserves, good economy, low density (2.70 g cm^−3^), fine processability, and reasonable thermal conductivity (217 W m^−1^ K^−1^) [8,9,10]. For advanced electronic packaging material, the TC of aluminum needs to be further improved. Additionally, the coefficient of thermal expansion of aluminum is too high (23.2 × 10^−6^ K^−1^) to match the common semiconductor materials. High-quality diamonds, which have an ultrahigh TC of about 1000–2200 W m^−1^ K^−1^, are regarded as excellent TC reinforcements [7,11]. Moreover, the coefficient of thermal expansion of diamond is only about 2.3 × 10^−6^ K^−1^. Recently, Al/Diamond composites, with their excellent heat dissipation performance and low coefficient of thermal expansion, have been regarded as promising to break through the heat dissipation bottleneck of high-power density electronic devices, which has triggered extensive attention and research reports [12,13,14,15].

Currently, there are various approaches to preparing Al/Diamond composites, including spark plasma sintering (SPS) [16,17,18], squeeze casting [19], gas pressure infiltration [20], cold spray additive manufacturing [21], hot pressing [22], etc. In the manufacture of composites, the conventional liquid infiltration method heats the aluminum above its melting point and then allows it to fully coat the diamond particles. However, this path inevitably leads to the formation of brittle Al_4_C_3_ at the interface, which has been proven to degrade thermal conductivity severely by impeding heat transfer [23]. Powder metallurgy (PM) methods, which can consolidate diamond and Al powders at temperatures below the melting point of Al, are supposed to be able to effectively avoid the formation of Al_4_C_3_ at the interface [4,5,24,25]. SPS is a mainstream powder consolidation technique widely applied to prepare Al/Diamond composites, which utilizes a pulse electric current via the powder to generate a localized heat flow that only partially softens or melts the particles for consolidation. Due to the low heating temperature (only 520–600 °C). and short holding time (several minutes) of this technology, the formation of Al_4_C_3_ at the interface can be effectively avoided, which is conducive to improving the TC of the Al/Diamond composite material [1,20,23,25,26,27]. However, SPS furnaces are typically equipped with complex and expensive pulsed power supplies and control systems, which result in bulky and expensive equipment with limitations on industrial applications. The demand for new technologies and equipment for powder metallurgy sintering remains significant.

Herein, a novel direct current (DC)-assisted fast hot-pressing (FHP) powder sintering technique developed by our group was utilized to prepare Al-50Diamond composites (the volume ratio of the diamond is 50%). The FHP method achieves ultra-high heating rates comparable to the SPS technique with an affordable DC power supply rather than the complex and expensive pulsed power supply of the SPS furnace. The FHP sintering furnace satisfies the special needs of Al/Diamond powder sintering with a greatly simplified plant design, reducing its cost to one-quarter of that of the SPS sintering furnace. Three series of orthogonal experiments were designed and conducted to explore the effects of sintering temperature, sintering pressure, and holding time on TC and sintering mechanism of Al-50Diamond composite. Multiple thermal conductivity models were employed to predict the TC of Al-50Diamond prepared by the FHP approach with optimized sintering parameters, as well as to investigate the effect of interfacial thermal resistance on TC performance.

## 2. Experimental Process

### 2.1. Materials

**Figure 1:**
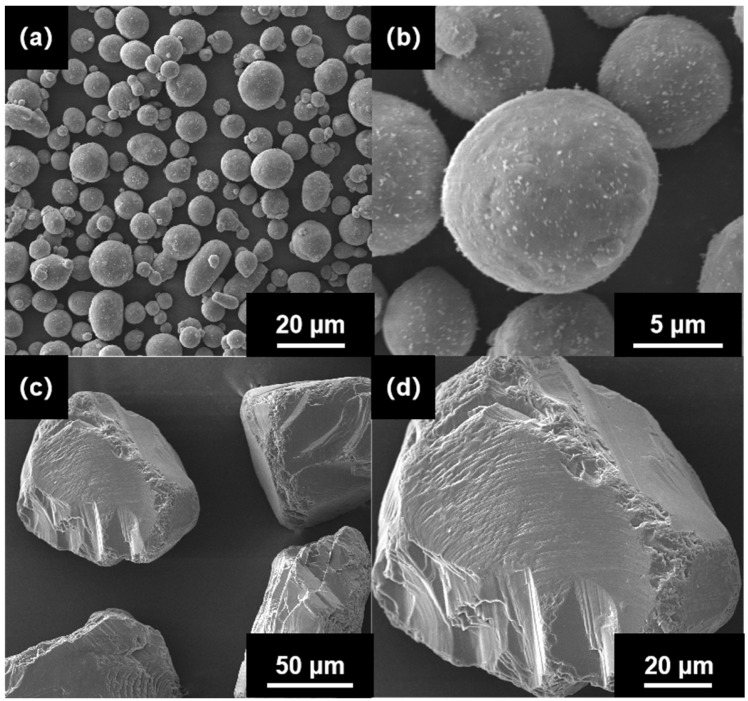
SEM images of (**a**,**b**) Al powders and (**c**,**d**) diamond powders with different magnifications.

**Figure 2:**
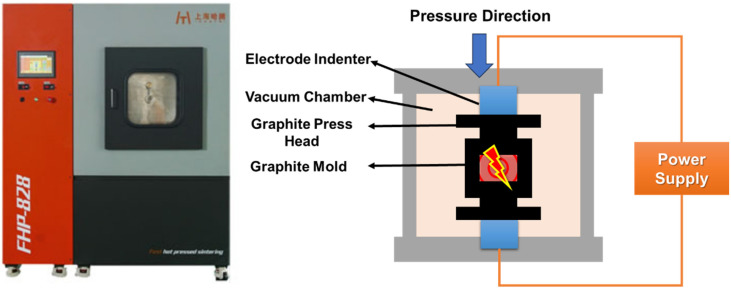
Core working parts of FHP-828 fast hot-pressing sintering furnace. (Figure 2 comes from our earlier work of Ref. [28]).

The Al-50Diamond composite comprised pure Al powder (~20 μm, provided by SCM Metal Products Co., Ltd., Suzhou, China) that was mixed with MBD4 diamond (~80 μm, provided by New United Diamond Technology Co., Ltd., Zhecheng, China) by the Fast Hot-Pressing (FHP) method (Figure 1). The pure Al and diamond powders were sintered via a fast hot-pressing sintering furnace (FHP-828, Haatn Technology Co., Ltd., Suzhou, China), as shown in Figure 2 (Figure 2 comes from our earlier work of Ref. [28]). The core of the FHP-828 furnace consists of a high-voltage DC power supply, a vacuum chamber, a pair of electrode indenters, and a graphite mold (or hard alloy). The Al powder and diamond powder were first mixed homogeneously at a volume ratio of 1:1 to obtain the composite precursor by a vibrating mixer (Haatn Technology Co., Ltd., Suzhou, China). The composite precursor was successively transferred into the graphite mold, and the mold was placed in the FHP furnace chamber. After the air was evacuated to a 10 Pa vacuum, the composite precursor was heated from 15 °C to 500 °C at a fast-heating rate of 100 °C min^−1^ and then heated to the sintering target temperature at a slow heating rate of 10 °C min^−1^ with holding for a certain amount of time at constant pressure. Eventually, Al-50Diamond composite samples with a disk shape were obtained by furnace cooling to room temperature. The samples were then cut into regular shapes and polished before successive characterization.

In order to investigate the effect of the preparation process on the density and thermal conductivity of Al-50Diamond by the FHP method, three series of orthogonal experiments were designed and carried out to adjust the sintering temperature, sintering pressure, and holding time, respectively. The specific parameter settings are shown in Table 1.

### 2.2. Material Characterization

**Figure 3:**
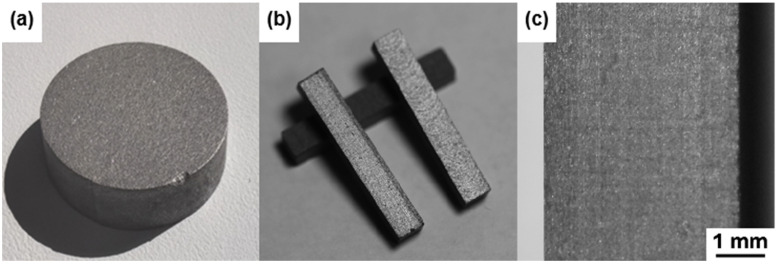
Optical photographs of Al-50Diamond composite specimens. (**a**) round disk sample, (**b**) samples cut into cuboid shape, (**c**) surface of the polished surface.

The Al-50Diamond composite specimens require surface polishing before characterization. A sandblaster was applied to remove the graphite paper stuck to the surface of the specimen. Subsequently, the specimens were polished with 400, 800, and 1200 mesh SiC sandpaper, followed by the mechanical polishing with diamond paste W2.5 (4000 mesh, Ra < 0.025 μm) for 10 min at a speed of 1000 RPM (YMPZ-2-300, Shanghai Metallurgical Equipment Co., Ltd., Shanghai, China). Al-50Diamond composite disk specimens with 50 vol.% diamond for characterization were obtained. Typically, the samples are round disks (with a diameter of 20 mm in Figure 3a, for example). For different characterizations, the composite specimens were cut into other shapes (Figure 3b). Additionally, in the macroscopic view, the polished surfaces are generally uniform with a slightly granular sensation (Figure 3c).

The real densities of samples were measured using a densitometer (PMDT AR-150PM, Dongguan Hongtuo Equipment Co., Ltd., Dongguan, China). The relative density is calculated by dividing the real density by the theoretical density based on the densities of pure Al and diamond. The microscopic morphology was observed by scanning electronic microscopy (SEM, S-4800, Hitachi, Tokyo, Japan). The surface Vickers hardness was measured by a Vickers hardness tester (YZHV-1000B, Shanghai Yizong Precision Instrument Co., Ltd., Shanghai, China).

Prior to measuring the thermal conductivity (TC, W m^−1^ K^−1^) of the Al-50Diamond composite, both the upper and lower surfaces of the specimens were sprayed with carbon to ensure that the temperature signals emitted from different surfaces were consistent. The thermal conductivity is calculated by Equation (1) as follows:

(1) $$\mathrm{TC}=\alpha \rho_{r}C_{p}$$

where α is the thermal diffusivity (m^2^ s^−1^) measured by laser flash analyzer (LFA 467 HyperFlash, NETZSCH Analyzing & Testing, Selb, Germany), C_p_ is the specific heat capacity (J kg^−1^ K^−1^)) measured by differential scanning calorimeter (DSC 214, NETZSCH Analyzing & Testing, Germany), and ρ_r_ is the real density of Al-50Diamond composite disk specimen with 50 vol.% diamond measured by densitometer.

## 3. Experimental Results and Discussion

### 3.1. Effect of Parameter Optimization of FHP Sintering Process on Microstructure and Thermal Conductivity

In order to elucidate the working principle of the FHP sintering process, the Al-50Diamond composite was selected to explore the critical factors that affected the performance of sintering. Therefore, the sintering temperature, pressure, and holding time of FHP sintering process were investigated and optimized by univariate controlled method, respectively.

#### 3.1.1. Sintering Temperature

The interfacial bonding between diamond particles and Al matrix is directly influenced by the wettability between them. Increasing the sintering temperature can effectively reduce the viscosity and surface tension of liquid-phase Al for better bonding with diamond particles [29]. However, excessively high temperatures can cause an overflow of the liquid-phase Al from the mold under high pressure and the following obvious agglomeration of diamond particles, which are also unfavorable to the interfacial bonding of the composites and the principle of energy saving. Therefore, an appropriate sintering temperature needs to be explored for these Al-50Diamond composites.

**Figure 4:**
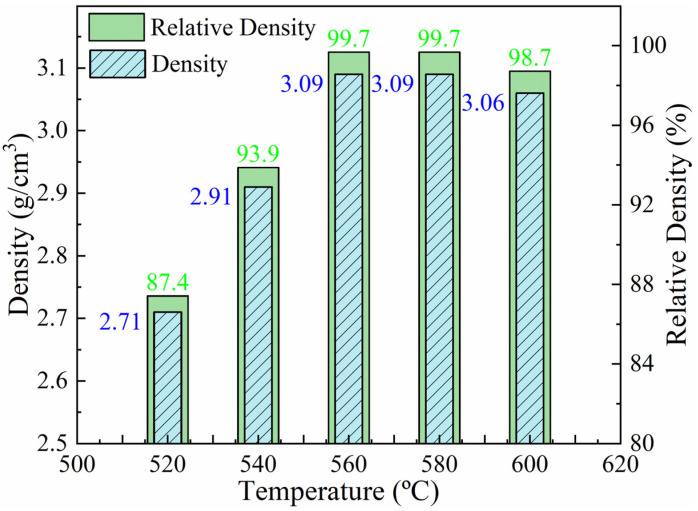
Density variation in Al-50Diamond composites at different sintering temperatures (the sintering pressure is 80 MPa, and the holding time is 10 min).

The densities of the Al-50Diamond composites prepared by FHP sintering at 520 °C, 540 °C, 560 °C, 580 °C, and 600 °C are presented in Figure 4 (the sintering pressure was set at 80 MPa, and the holding time was set at 10 min, uniformly). The results present a general increasing density of the composites with sintering temperature from 520 °C to 560 °C. The corresponding densities vary from 2.70 g cm^−3^ to 3.09 g cm^−3^. The maximum density is 3.09 g cm^−3^ (99.7% of the theoretical density of Al-50Diamond composite) obtained at 560 °C and 580 °C, respectively. Continuing to increase the sintering temperature to 600 °C, the density of the composite turns to decrease gradually. This phenomenon can be explained in the following discussions.

The melting point of the diamond is much higher than that of the Al matrix; thereby, the viscosity and fluidity of the Al matrix determine the size and generation rate of pores in the composite. The Al matrix becomes soft at about 300 °C, and as the temperature increases, the plastic deformation ability of the Al matrix increases. In addition, the wettability between Al and diamond particles is not good. At the lower sintering temperature (≤560 °C), the plastic deformation ability of Al is not strong enough. It results in the insufficient plastic deformation of Al, and a higher number of pores remain between the two phases of the composite, which ultimately causes the density of the composite to be significantly lower than the ideal density. However, as the temperature increases to 580 °C or even higher, the density of the composites begins to decrease slowly. The excessive drop in the viscosity of the Al leads to debonding and cracking at the Al/diamond interface due to the bad wettability and stacking of those non-deformable diamond particles to form voids, causing the decrease in the density. Only if the sintering temperature remains in a suitable range (560–580 °C) can porosity and debonding cracks be effectively minimized, realizing the maximum density of the sintered composite [30,31,32].

**Figure 5:**
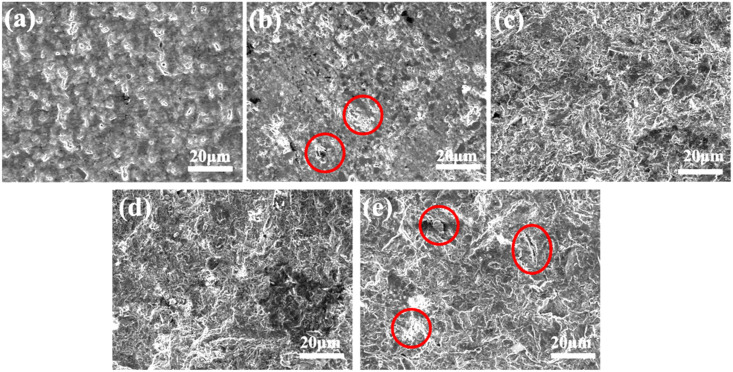
The microstructures of Al-50Diamond composites at (**a**) 520 °C, (**b**) 540 °C, (**c**) 560 °C, (**d**) 580 °C, and (**e**) 600 °C sintering temperatures (the sintering pressure is 80 MPa, and the holding time is 10 min).

The microstructures of Al-50Diamond composites at different sintering temperatures (520–600 °C) obtained via SEM confirm the speculation above (Figure 5). As shown in Figure 5a,b, there are obvious pores on the cross-section of the samples sintered at 520 °C and 540 °C, respectively. This is because, at the relatively low sintering temperature (≤560 °C), the Al phase is not able to sufficiently encapsulate the diamond particles, leaving the diamond alone exposed (red circles in Figure 5b). Similarly, the higher sintering temperature (≥580 °C) caused debonding cracks (red circles in Figure 5e). In contrast, the pores and debonding cracks could be effectively reduced or even prevented by suitable sintering temperatures (560–580 °C, Figure 5c,d).

**Figure 6:**
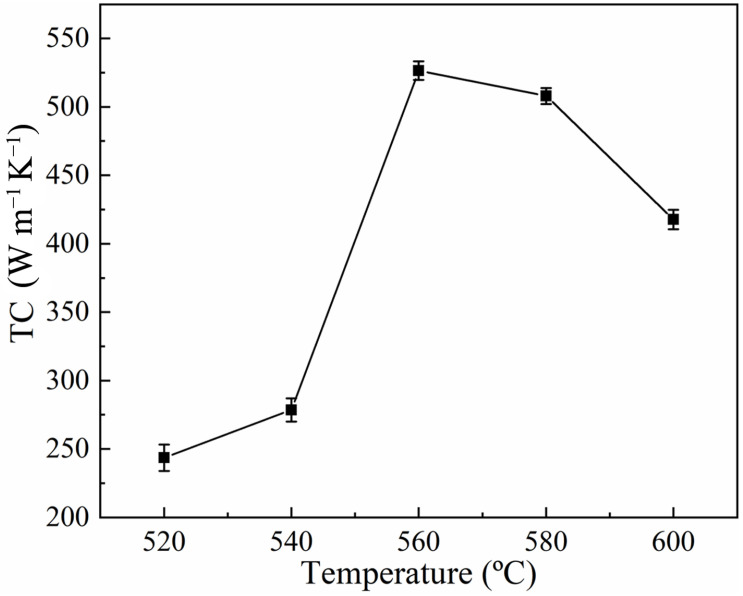
Thermal conductivity of Al-50Diamond composites at different sintering temperatures (the sintering pressure is 80 MPa, and the holding time is 10 min).

Figure 6 exhibits the TC of Al-50Diamond composites prepared at different sintering temperatures. The TC of Al-50Diamond composites tends to increase sharply to reach a maximum value and then decrease slowly, which is basically consistent with the trend of density variation (Figure 4). In conventional composites, the interfacial thermal resistance generated between the reinforcement particles and the matrix is a crucial factor affecting its TC [3,26,33,34]. As discussed above, the sintering temperature effectively affects the appearance of the pores and debonding cracks, which produces the interfacial space and enhances thermal resistance. At relatively low sintering temperatures (520–540 °C), the samples with pore structures possess deteriorated interfacial bonding conditions and TC (less than 278.5 W m^−1^ K^−1^). The possibility of debonding cracks is dramatically increased again as the sintering temperature increases to 580 °C or above, which opposes reducing interfacial thermal resistance (506.9 W m^−1^ K^−1^ or less). Consistent with the variation in density, the TC of the composites reaches a maximum value (527.8 W m^−1^ K^−1^) at a sintering temperature of 560 °C.

#### 3.1.2. Sintering Pressure

**Figure 7:**
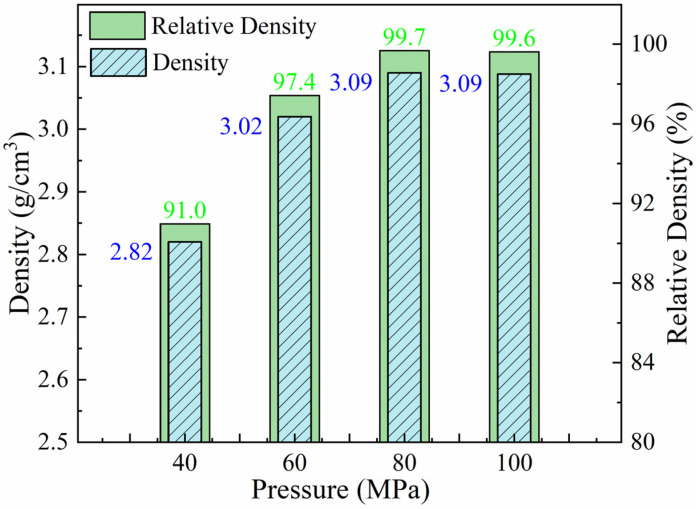
Density variation in Al-50Diamond composites at different sintering pressures (the sintering temperature is 560 °C, and the holding time is 10 min).

Sintering pressure provides the driving force for plastic deformation of the Al matrix, and it is inevitable that sintering pressure would influence the microstructure and TC of Al-50Diamond composites, just like sintering temperature. On the basis of the determined optimal sintering temperature, the effect of sintering pressure on the conformal materials will be discussed next. The sintering pressure gradually increased (40 MPa, 60 MPa, 80 MPa, and 100 MPa) at a fixed sintering temperature and holding time (560 °C and 10 min). Figure 7 demonstrates the density variation in Al-50Diamond composites at different sintering pressures. With the increase in sintering pressure, the density of the samples shows an increasing and then slightly fluctuating trend, where the maximum value of 3.09 g cm^−3^ was reached at 80 MPa (99.7% relative density). During the process of increasing the sintering pressure, lower sintering pressure (≤60 MPa) could not provide sufficient driving force to make the Al plastically deform and encapsulate the diamond particles, causing the density of the composites to be much less than the theoretical density. When the critical pressure was reached and exceeded (80 MPa), the Al was able to plasticly deform sufficiently and completely encapsulate the diamond particles, reducing the incidence of pores and debonding cracks, ultimately resulting in a composite density close to the theoretical density. A further increase in sintering pressure (≥100 MPa) thereafter resulted in almost no change in the density of the composites.

**Figure 8:**
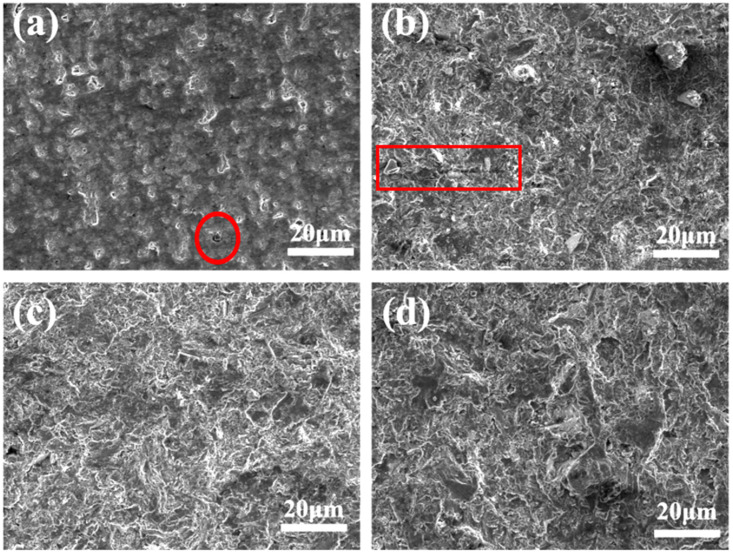
The microstructures of Al-50Diamond composites at (**a**) 40 MPa, (**b**) 60 MPa, (**c**) 80 MPa, and (**d**) 100 MPa sintering pressures (the sintering temperature is 560 °C, and the holding time is 10 min).

The microstructures of Al-50Diamond composites at different sintering pressures (40–100 MPa) agree with the densities very well (Figure 8). Figure 8a indicates that when the sintering pressure is 40 MPa, much lower than 80 MPa, the Al matrix does not completely enclose the diamond particles, resulting in obvious pores in the microstructure (red circle). As sintering pressure increases to 60 MPa, the number of pores decreases dramatically, but a small number of transgranular cracks (red rectangle) still remains (Figure 8b). The phenomena indicate that increasing the sintering pressure could effectively promote interfacial bonding between the Al phase and diamond particles. Pores and cracks can be almost completely eliminated at an appropriate sintering pressure of 80 MPa (Figure 8c). However, extremely high pressure (≥100 MPa) would trigger plastic deformation and produce high-density dislocations, which significantly increases the possibility of defects [15,35] (Figure 8d).

**Figure 9:**
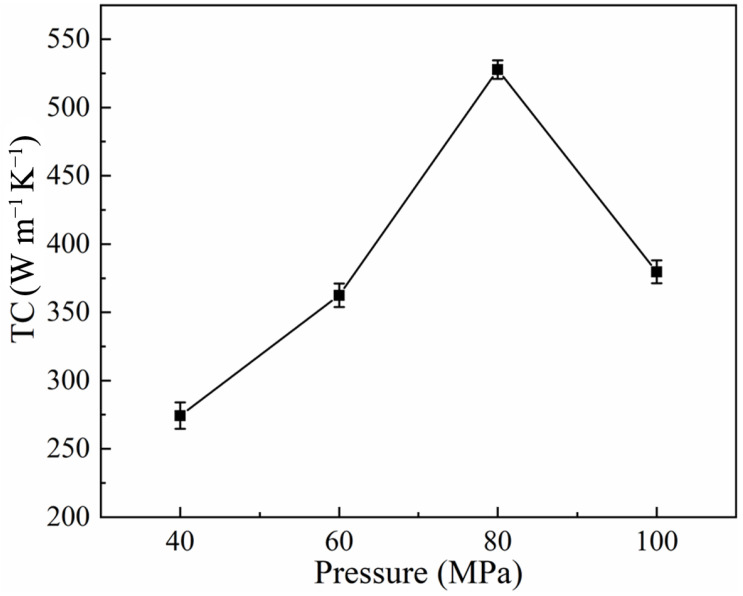
Thermal conductivity of Al-50Diamond composites at different sintering pressures (the sintering temperature is 560 °C, and the holding time is 10 min).

The variation curve of TC with sintering pressure for Al-50Diamond composites is shown in Figure 9. The TC of the composites shows a rapid increase to a maximum value (527.8 W m^−1^ K^−1^) and then a significant decrease with the increase in sintering pressure. Compared with the microstructure shown in Figure 8, the interfacial bonding between the Al matrix and diamond particles is enhanced with the increase in sintering pressure before 80 MPa, which reduces interfacial thermal resistance and ultimately improves the TC performance. However, excessive sintering pressure (100 MPa) would induce obvious plastic deformation ability of the Al matrix to produce defects like stacking of diamonds, which increases the interfacial thermal resistance and degrades TC performance. Therefore, during the sintering process, the magnitude of the applied pressure needs to be precisely controlled to provide enough pressure to fully bond the Al matrix and diamond particles while avoiding excessive pressure that would cause the stacking of diamond particles and defects in the microstructure. To summarize the above discussion, the optimum sintering pressure for Al-50Diamond composites is 80 MPa under the parameters of 560 °C and 10 min of heat preservation.

#### 3.1.3. Holding Time

**Figure 10:**
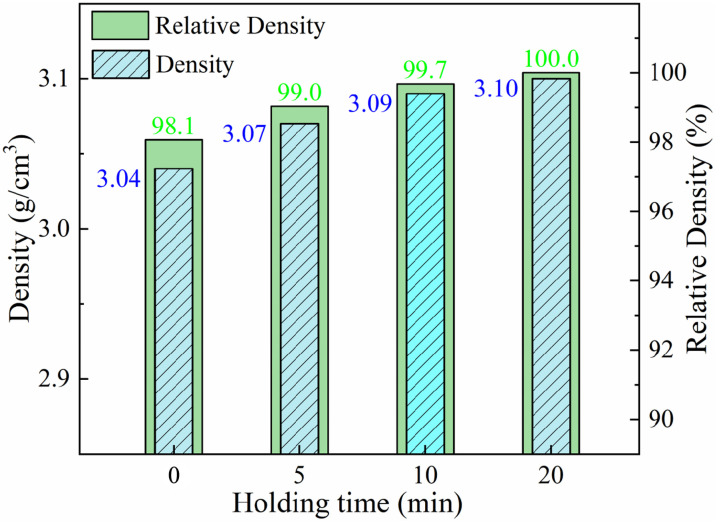
Density of Al-50Diamond composites at different sintering holding times (the sintering temperature is 560 °C, and the sintering pressure is 80 MPa).

The plastic deformation and fusion of Al particles would surely take a certain period of time, as well as the bonding between Al and diamond. The exploration of optimal sintering time in this section will continue based on the previous two subsections. The sintering temperature and sintering pressure were fixed at 560 °C and 80 MPa, while the holding times were set as 0 min, 5 min, 10 min, and 20 min, respectively. The corresponding results about density are presented in Figure 10. The general trend is a monotonic increase in sample density with increasing holding time. The densities of the prepared composites are all greater than 99.7% when the holding time was higher than 10 min under the sintering conditions of 560 °C and 80 MPa.

**Figure 11:**
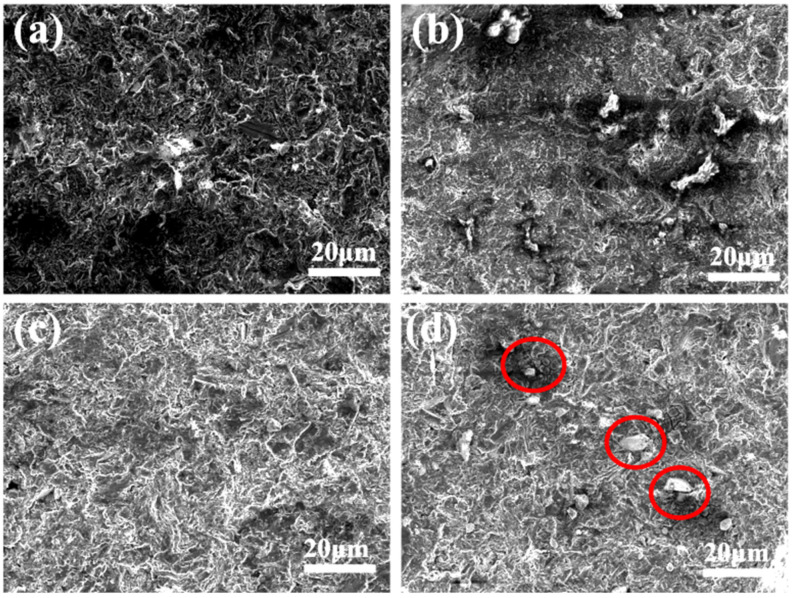
The microstructures of Al-50Diamond composites sintered at 560 °C-80 MPa for (**a**) 1 min, (**b**) 5 min, (**c**) 10 min, and (**d**) 20 min, respectively (the sintering temperature is 560 °C and the sintering pressure is 80 MPa).

**Figure 12:**
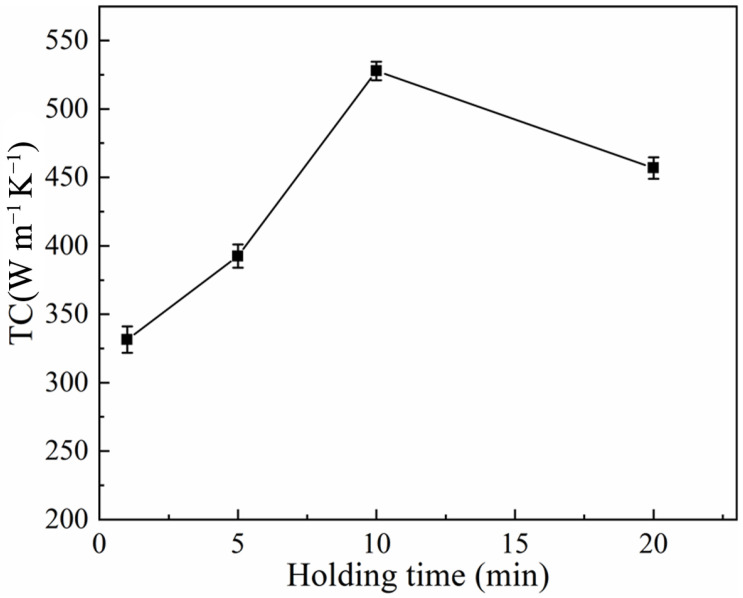
Thermal conductivity of Al-50Diamond composites at different holding times (the sintering temperature is 560 °C, and the sintering pressure is 80 MPa).

Figure 11 illustrates the microstructures of Al-50Diamond composites sintered at 560 °C-80 MPa for different holding times. For insufficient holding times (≤5 min), the liquid-phase Al does not spread completely into the pores before it starts to cool down and condense, which results in the density of the composite being lower than the theoretical density (Figure 11a). By extending the holding time to 20 min, the diamond particles in the composites are exposed, with significant interfacial debonding between them and the surrounding Al matrix (red-circled regions in Figure 11d). Only proper hold time leads to a uniform cross-section, which benefits the TC of Al-50Diamond composites (Figure 11c). The results indicate that the interfacial bonding strength has a minimal effect on the density of the Al-50Diamond composite but strongly affects the TC of the material. Similar phenomena are also observed in the conventional SPS sintering method [1,25,28,32,36]. Figure 12 presents the variation curve of thermal conductivity with holding time for the Al-50Diamond composite. The overall TC trend exhibits a mountainous shape, with a maximum value present at a given holding time (10 min for Al-50Diamond composites at 560 °C-80 MPa sintering condition). Al-50Diamond composites are sintered at 560 °C-80 MPa for 10 min to achieve a maximum TC of 527.8 W m^−1^ K^−1^. The presence of porosity and interfacial debonding gaps in the microstructure strongly degrade the TC of the composites.

**Figure 13:**
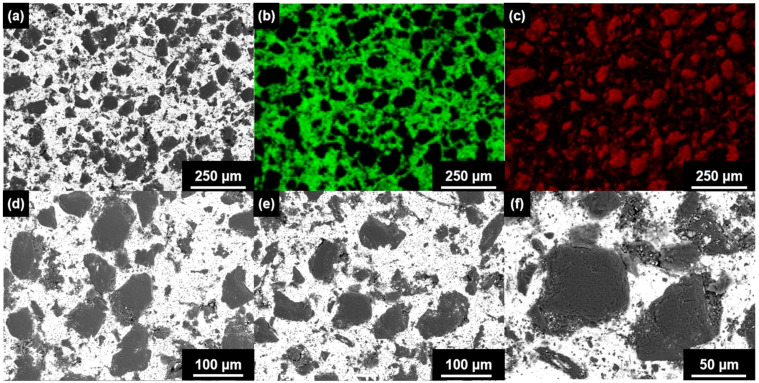
(**a**,**d**–**f**) SEM images in BSE contrast of Al-50Diamond composite disk prepared at 560 °C-80 MPa-10 min with different magnifications and the EDS mapping images and mapping images of Al ((**b**), green) and C ((**c**), red) for (**a**).

In order to further comprehend the relation between TC and microstructure, the SEM images in BSE contrast and EDS mapping images of the Al-50Diamond composite disk prepared at 560 °C-80 MPa-10 min were obtained after cutting and polishing (Figure 13). As can be seen, a number of irregular diamond particles (red region in Figure 13c) distribute homogeneously in the Al continuous matrix (green region in Figure 13b). In the high-resolution SEM images (Figure 13d–f), the Al matrix and diamond particles are closely connected, and no obvious cracks could be observed. This compact structure contributes to the best thermal conductivities.

**Figure 14:**
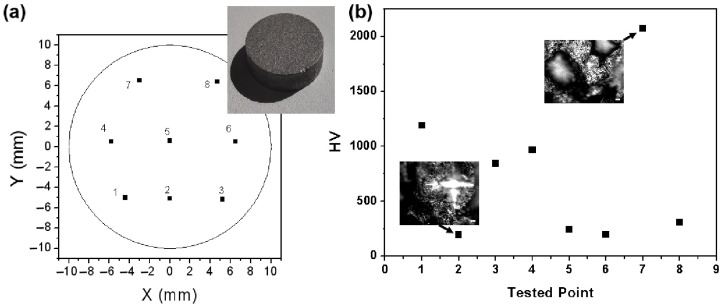
(**a**) Distribution of surface Vickers hardness tested points of Al-50Diamond composite disk with the diameter of 20 mm prepared at 560 °C-80 MPa-10 min and (**b**) the surface Vickers hardness of different tested points.

**Figure 15:**
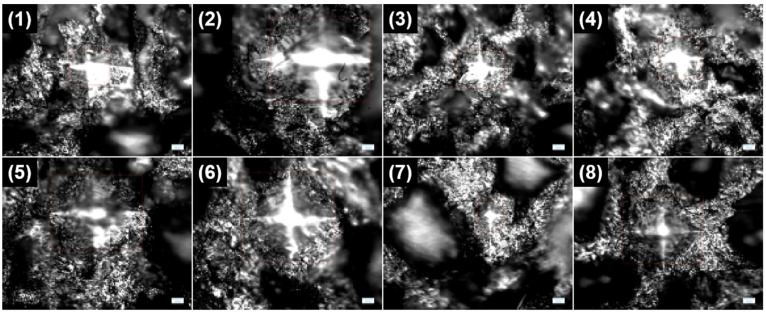
Optical images of the surface Vickers hardness tested points in Figure 14.

Furthermore, we also investigate the surface Vickers hardness of the Al-50Diamond composite disk prepared at 560 °C-80 MPa-10 min. Eight random points are selected (Figure 14a), and the values of Vickers hardness for these eight points differ greatly. The smallest value is 195.4 HV, while the highest value reaches as much as 2070.9, more than ten times the smallest value (Figure 14b). The optical images of the indentation provide more information (Figure 15). In the optical images, two kinds of regions with different reflections can be observed: the continuous region should be Al, and the particle region should be diamond based on Figure 13. When there are no diamond particles near the indentation, like Points 2, 5, 6, and 8, the surface Vickers hardness is relatively small. On the contrary, when the diamond particles are quite near the indentation, like Point 7, the surface Vickers hardness is extremely high. For other conditions, the diamond particles are not too far from or too near the indentation, like Points 1, 3, and 4, and the surface Vickers hardness is medium. In summary, the distribution of surface Vickers hardness also agrees with the distribution of diamond particles in the Al matrix observed in Figure 13.

### 3.2. Sintering Mechanism of Al/Diamond Composites Prepared by FHP Process

The techniques of sintering bulk composites by current and mechanical pressure, represented by the SPS method [18,25,28,37], have been widely utilized in the field of powder metallurgy in recent years. These sintering methods have two major advantages. On one hand, it requires lower sintering temperatures and provides higher sintering efficiencies, as compared to conventional methods such as non-pressurized sintering and hot-pressurized sintering. On the other hand, these methods can be used to prepare special structural composites, including functional gradient structures. For the preparation of Al-50Diamond composites, the optimal temperature of the conventional air pressure permeation method is as high as 750 °C, while the value of the FHP method involved in this paper is only 560 °C. The TC of the Al-50Diamond composite prepared by the former is 514.7 W m^−1^ K^−1^, which is slightly lower than that of the latter, 527.8 W m^−1^ K^−1^. Furthermore, the FHP device can significantly shorten the sintering process of Al-50Diamond composites to less than 20 min by far exceeding the heating rate of conventional devices by up to 100 °C min^−1^. The FHP method can significantly shorten the production cycle and achieve substantial energy savings without deteriorating the thermal conductivity, which is promising for commercial production. An illustration of the sintering mechanism of the FHP method will be given as an example of an Al-50Diamond composite in the following discussion.

**Figure 16:**
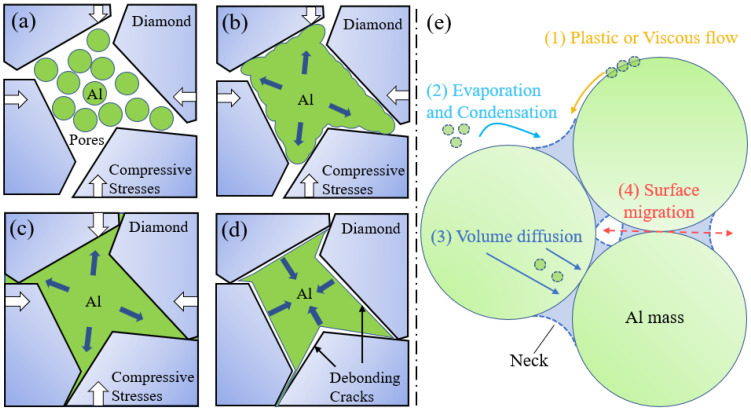
Schematic illustration of the FHP method in preparing Al/diamond composites. (**a**) Porous state of Al/diamond powder after loading into graphite molds. (**b**) Mass transport of partially molten Al particles under direct current, heating, and pressurized conditions. (**c**) Ideal Al/diamond composites prepared under the appropriate conditions. (**d**) The phenomenon of debonding occurs after cooling to room temperature as a result of excessively high sintering temperatures or excessively long holding times (White bold arrows means pressure). (**e**) Four possible mass transport mechanisms in sintering processes.

Sintering is a technique that reduces inter-particle voids by decreasing the surface curvature of the powder particles to achieve densification. For any irreversible physical or chemical reaction, lowering the free energy of the system is the first step of the process. Specifically for the FHP sintering process, the driving force to reduce the free energy of the composite consisted of two components: the applied pressure and the surface tension of the melt or the softened material [28,30,31]. During sintering heating and holding, the combined effect of pressure and surface tension reduced the surface curvature of the molten particles and excluded porosity to achieve densification. Figure 16 presents a schematic diagram of Al/Diamond composites prepared by the FHP method. Figure 16a describes the initial porous state of the Al/diamond powder after loading it into graphite molds. Despite applying enormous pressure to achieve compact contact between the matrix particles, higher stiffness of the Al/diamond materials would have severely resisted plastic deformation, resulting in the retention of a large number of porosities. Therefore, in order to extrude the pores by plastic deformation of the composite powder under compressive stresses (Figure 16b), a sufficiently high-temperature environment (≥560 °C, Figure 3) must be provided to melt or soften some of the Al particles’ surfaces to decrease the stiffness and surface tension. An alternative approach to generating plastic deformation under the condition that the size and shape of the particles remained constant was to further increase the applied stress (≥80 MPa, Figure 6) to forcefully overcome the matrix stiffness and surface tension. The effect of holding time on the densification of the composites was not significant and showed a slow linear positive correlation (Figure 9). Figure 16c describes that the ideal sintered composite should be a uniformly dispersed system free of porosity under suitable temperature, pressure, and holding time. Otherwise, the debonding cracks would occur due to unsuitable sintering parameters (Figure 16d), e.g., extremely high temperature (≥600 °C). This could be attributed to the considerable difference in the coefficient of thermal expansion (CTE) between Al (23.6 × 10^−6^ K^−1^) and diamond (2.3 × 10^−6^ K^−1^) [38]. The large thermal stress generated at the interface on diamond particles exhibited as compressive stress during the heating process and tensile stress during the cooling process. During heating, compressive stresses can be continuously released via the molten or softened aluminum, filling the rest of the space enclosed by the particles. However, during cooling, tensile stresses instead cause the interface between the aluminum and diamond to shrink and debond. As shown in Figure 16e, there are four possible mechanisms for mass transport during sintering: (1) plastic or viscous flow, (2) evaporation and condensation, (3) volume diffusion, and (4) grain boundary migration. At lower sintering temperatures, the surface tension of a solid metal is undoubtedly of the same order of magnitude as that of a molten metal, i.e., 10^3^ erg/cm^2^ [28]. The free diffusion drive of most Al atoms was not sufficient enough to completely escape the surface tension of the particles, which limited the plastic or viscous flow of the atoms along the particle surface (≤540 °C, Figure 5b) [39,40]. During the FHP sintering process, the DC induces the directional movement of metal electrons on the surface of the Al particles to generate Joule heating and localized high temperatures, which promotes surface softening or melting, expansion, and adhesion of the Al powder particles to the diamond particles. As the sintering temperature increased (≥560 °C), more exterior Al melted to form the liquid phase, and the plastic deformation ability of Al is promoted, which continuously fills the interstices of the diamond particles, resulting in densification. Simultaneously, more atoms escaped interatomic attraction and surface tension (evaporation) [40], with the possibility of being adsorbed by the interface. The energy of the vapor atoms was much higher than the energy of the atoms in the adsorption interface. As a result, the newly adsorbed adatoms equipped with excess energy break the equilibrium state of the interfacial atoms. It has been demonstrated that high-energy adsorbed adatoms spontaneously undergo directed surface diffusion into low-energy regions (e.g., pores) to achieve densification [28,39,40]. Directional surface diffusion of atoms into the pores reduces the surface curvature of the particles. Due to the presence of surface tension, adatoms are more inclined to connect different interfaces to form a necking structure rather than simply being adsorbed at a single surface to coarsen the particles. After the interfacial connection of different particles, volume diffusion is triggered by the existence of thermal difference, eventually resulting in the surface migration to form a gourd-like or mitochondria-like structure.

### 3.3. Thermal Conductivity of Al/Diamond Composites Prepared by FHP Process

Thermal conductivity is the primary consideration for Al/diamond composites used in high-performance processor heat sinks. The theoretical thermal conductivities of Al and diamond are 217 W m^−1^ K^−1^ and 1800 W m^−1^ K^−1^ [11], respectively. Naturally, the TC of the composite decreases as the percentage of Al increases. In the discussion of this subsection, the difference between the Al-50Diamond composite prepared by the FHP method and the theoretical TC is compared on the basis of fixing the volume fraction of Al at 50 vol.%, followed by an analysis of the factors responsible for this difference.

The theoretical TC of binary solid-phase composites (***K***) is usually considered to be a function of the TC of the continuous matrix phase (***k_m_***), the TC of dispersed particle phase (***k_p_***), the volume fraction of the particle phase (***V***), the shape and arrangement of the particle phase (***α***), etc. The theoretical function of TC usually takes the following form [41,42]:

(2) $$K=K(k_{m},k_{c},V,\alpha )$$

**Figure 17:**
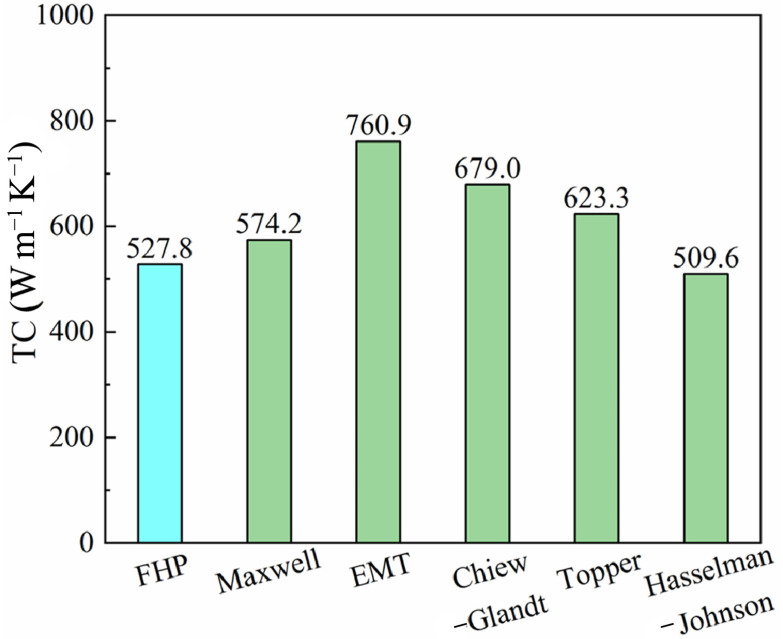
Thermal conductivities of Al-50Diamond composite: FHP tested values and theoretical values based on different models.

Since thermal conductivity is affected by numerous factors, a number of researchers have been working on modeling TC for different systems. In the case of the Al-50Diamond composite, herein, the following widely utilized theoretical and empirical models are adopted, including the Maxwell model [7,43], Effective medium theory (EMT) model [7,44], Chiew–Glandt model [7,45], Topper model [7,46], and Hasselman–Johnson Model [7,26]. The models and formulas involved in calculating theoretical thermal conductivity are summarized in Table 2. The results in Figure 17 show that the predictions of each model are much higher than the actual test values of the FHP sintering sample. The classic Maxwell model considers the problem of dilute dispersion of spherical particles (diamond) embedded in a continuous matrix (Al). Without considering the interfacial differences and thermal resistances, the calculated value of 574.2 W m^−1^ K^−1^ from the classical Maxwell model shows a deviation of 8.0% from the actual measured value of 527.8 W m^−1^ K^−1^ for the FHP sintered sample. The EMT, Chiew–Glandt, and Topper models’ theoretically calculated values differ even more from the actual measured values and cannot effectively predict the TC of the Al-50Diamond composite. The large deviation of the theoretically calculated value of TC from the actual measured value can be attributed to the interfacial thermal resistance [1,33], which is the reciprocal of interfacial thermal conductivity.

When heat flows through the particle or phase interface of a composite material, a sudden drop in temperature will be observed at the interface. This discontinuity in heat flow can be described in terms of thermal resistance. It is called interfacial thermal resistance (ITR) and consists of the combined effect of two thermal resistances. The first is an intrinsic property of the composite material known as thermal contact resistance (TCR), which is caused by inadequate mechanical and chemical bonding between the constituent phases. The latter is thermal boundary resistance (TBR), which results from differences in the physical properties of the constituent materials and is also known as Kapitza resistance [33,36]. Differences in vibrational and electronic properties between two distinct solid phases cause resistance to thermal convection resistance at the boundary. Heat carriers (phonons or electrons) reaching the interface will be scattered, and the probability of transmission after scattering depends on the available energy states on both sides of the interface [33]. Therefore, all factors that are unfavorable to heat carrier scattering, such as pores, dislocations, debonding, etc., significantly increase the ITR, resulting in a decrease in the TC of the Al-50Diamond composites [1,11,16,36,38,47]. Hasselman and Johnson [26] emphasized the importance of ITR and proposed a simple modification of the original Maxwell and Rayleigh models to derive the first expressions for effective TC of composite materials with non-zero ITR, as shown in Table 2. Considering the interfacial thermal resistance, the theoretical TC calculated by the Hasselman–Johnson formula is 509.6 W m^−1^ K^−1^, which is 3.4% lower than the actual TC of the FHP sintered samples (527.8 W m^−1^ K^−1^). Therefore, the Hasselman–Johnson formula incorporating the interfacial thermal resistance theory can excellently explain and predict the thermal conductivity of FHP sintered specimens in the presence of defects such as pores, thermal stress-induced dislocations, debonding cracks, etc.

To investigate the feasibility, reliability, and advancement of the FHP technique, the thermal conductivity of Al-50Diamond composites prepared by other researchers via VHP and SPS techniques are compared in Table 3. The TC of Al-50Diamond composites prepared by the FHP technique is comparable to that of composites with similar compositions prepared by the VHP technique and both are higher than the conventional SPS technique. There is a large difference in the TC of the same proportion of Al/Diamond composites prepared utilizing the same SPS sintering technique. This is due to the fact that the parameters such as temperature, pressure, and holding time during sintering are not optimally adjusted, resulting in excessive interfacial thermal resistance.

In conclusion, FHP is a novel sintering technique that can efficiently produce Al/Diamond composites by generating localized heat via DC current. The thermal conductivity of its samples can be comparable to sintering techniques such as VHP and SPS. Owing to its remarkable economy, the FHP technology will certainly be a promising route for the preparation of Al/diamond composites.

## 4. Conclusions

A novel DC-assisted fast hot-pressing (FHP) powder sintering technique was utilized to prepare Al-50Diamond composites. The optimized process parameters for the preparation of Al-50diamond composites by the FHP method were sintering temperature of 560 °C, sintering pressure of 80 MPa, and holding time of 10 min, corresponding to a density and TC of 3.09 g cm^−3^ (the relative density reaches 99.7%) and 527.8 W m^−1^ K^−1^, respectively, showing the Al-50Diamond composite possess great potential for thermal dissipation.

Several TC models are employed to calculate the TC, and some common models, such as the Maxwell model, are unable to accurately predict the actual TC of Al-50Diamond composites due to the non-negligible interfacial differences in Al and diamond. Taking ITR into account, the Hasselman–Johnson model can more accurately predict the TC of Al-50Diamond composites for FHP technology under optimal process parameters with a 3.4% error (509.6 W m^−1^ K^−1^ to 527.8 W m^−1^ K^−1^).

The TC of Al-50Diamond composites prepared by FHP technology is comparable to that of conventional powder sintering techniques (VHP and SPS). Under this premise, FHP technology has economic advantages such as fast heating rate and low sintering temperature, which is conducive to the popularization and application of this technology in the field of powder metallurgy.

## Figures and Tables

**Table 1:** Details of experiments for optimizing FHP sintering parameters for Al-50Diamond composite.

Experiment Series	Sintering Temperature (°C)	Sintering Pressure (MPa)	Holding Time (min)
Series A	520	80	10
	540		
	560		
	580		
	600		
Series B	560	40	10
		60	
		80	
		100	
Series C	560	80	0
			5
			10
			20

**Table 2:** The models and formulas involved in this paper for calculating theoretical thermal conductivity.

Model	Calculation Formula	Notes	No.
Maxwell Model	$K=k_{m}\cdot \frac{\kappa +2+2\left(\kappa -1\right)V}{\kappa +2-\left(\kappa -1\right)V}$	κ $\;\mathrm{is}\;\mathrm{the}\;\mathrm{ratio}\;\mathrm{of}\;k_{p}\;\mathrm{and}\;k_{m},$ $\kappa =\frac{k_{p}}{k_{m}}$	(3)
Effective Medium Theory Model	$K=k_{m}\cdot (\kappa A+\sqrt{\kappa^{2}A^{2}+\kappa /2})$	*A* is an empirical parameter, $A=\frac{1}{4}(3V-1+\frac{2-3V}{\kappa })$	(4)
Chiew-Glandt Model	$K=k_{m}\cdot \frac{1+2\beta V+\left(2\beta^{3}-0.1\beta \right)V^{2}+0.05exp(4.5\beta )V^{3}}{1-\beta V}$	*β* is the reduced thermal polarizability, defined as $\beta =\frac{\kappa -1}{\kappa +2}$	(5)
Topper Model	$\frac{1}{K}=\frac{1-V^{1/3}}{k_{m}}+\frac{V^{1/3}}{k_{p}V^{2/3}+k_{m}(1-V^{2/3})}$		(6)
Hasselman–Johnson Model	$K=k_{m}\cdot \frac{2(k-Rk_{p}/d-1)V+k+2Rk_{p}/d+2}{(1-k+Rk_{p}/d)V+k+2Rk_{p}/d+2}$	Considering the interfacial thermal resistance, *R* (2 × 10^−8^ K W^−1^ m^2^), which is the reciprocal of interfacial thermal conductivity measured by Stoner et al. [36] *d* is the average particle size of a diamond.	(7)

**Table 3:** Comparison of thermal conductivity of Al/Diamond composite in this paper and other Al/Diamond material at room temperature.

No.	Material	Processing Method	K (W m^−1^ K^−1^)	Note
1	Al-50Diamond	Vacuum Hot-Pressing (VHP)	496	Ref. [48]
2	Al-50Diamond	spark plasma sintering (SPS)	325	Ref. [30]
3	Al-45Diamond	SPS	403	Ref. [5]
4	Al-50Diamond	SPS	420	Ref. [27]
5	Al-50Diamond	FHP	527	This work

## Data Availability

The original data for this paper cannot be open sourced to the public due to the commercial confidentiality involved.
